# Asymmetry of radiomics features in the white matter of patients with primary progressive aphasia

**DOI:** 10.3389/fnagi.2023.1120935

**Published:** 2023-05-05

**Authors:** Benedetta Tafuri, Marco Filardi, Daniele Urso, Valentina Gnoni, Roberto De Blasi, Salvatore Nigro, Giancarlo Logroscino, on behalf of the Frontotemporal Lobar Degeneration Neuroimaging Initiative

**Affiliations:** ^1^Center for Neurodegenerative Diseases and the Aging Brain, University of Bari Aldo Moro, “Pia Fondazione Cardinale G. Panico”, Lecce, Italy; ^2^Department of Translational Biomedicine and Neuroscience (DiBraiN), University of Bari Aldo Moro, Bari, Italy; ^3^Department of Neurosciences, King’s College London, Institute of Psychiatry, Psychology and Neuroscience, London, United Kingdom; ^4^Sleep and Brain Plasticity Centre, CNS, IoPPN, King’s College London, London, United Kingdom; ^5^Department of Diagnostic Imaging, Pia Fondazione di Culto e Religione “Card. G. Panico”, Tricase, Italy

**Keywords:** primary progressive aphasia, radiomics, asymmetry, structural white matter, MRI, verbal fluency, neuroimaging

## Abstract

**Introduction:**

Primary Progressive Aphasia (PPA) is a neurological disease characterized by linguistic deficits. Semantic (svPPA) and non-fluent/agrammatic (nfvPPA) variants are the two main clinical subtypes. We applied a novel analytical framework, based on radiomic analysis, to investigate White Matter (WM) asymmetry and to examine whether asymmetry is associated with verbal fluency performance.

**Methods:**

Analyses were performed on T1-weighted images including 56 patients with PPA (31 svPPA and 25 nfvPPA) and 53 age- and sex-matched controls. Asymmetry Index (AI) was computed for 86 radiomics features in 34 white matter regions. The relationships between AI, verbal fluency performance (semantic and phonemic) and Boston Naming Test score (BNT) were explored through Spearman correlation analysis.

**Results:**

Relative to controls, WM asymmetry in svPPA patients involved regions adjacent to middle temporal cortex as part of the inferior longitudinal (ILF), fronto-occipital (IFOF) and superior longitudinal fasciculi. Conversely, nfvPPA patients showed an asymmetry of WM in lateral occipital regions (ILF/IFOF). A higher lateralization involving IFOF, cingulum and forceps minor was found in nfvPPA compared to svPPA patients. In nfvPPA patients, semantic fluency was positively correlated to asymmetry in ILF/IFOF tracts. Performances at BNT were associated with AI values of the middle temporal (ILF/SLF) and parahippocampal (ILF/IFOF) gyri in svPPA patients.

**Discussion:**

Radiomics features depicted distinct pathways of asymmetry in svPPA and nfvPPA involving damage of principal fiber tracts associated with speech and language. Assessing asymmetry of radiomics in PPA allows achieving a deeper insight into the neuroanatomical damage and may represent a candidate severity marker for language impairments in PPA patients.

## 1. Introduction

Primary Progressive Aphasia (PPA) is a rare neurological syndrome, with estimated prevalence of three cases per 100.000 people/persons in the general population (Coyle-Gilchrist et al., 2016). PPA is pathologically associated with Frontotemporal lobe degeneration (FTLD) and clinically characterized by prominent linguistic deficits (Mesulam, 1982; Gorno-Tempini et al., 2011; Leyton et al., 2011; Tee and Gorno-Tempini, 2019). PPA usually arises with subtle speech problems that could progress into an almost total inability to speak.

The semantic (svPPA) and non-fluent/agrammatic (nfvPPA) variants of primary progressive aphasia represent the two main clinical subtypes of PPA (Gorno-Tempini et al., 2011). svPPA and nfvPPA exhibit distinct patterns of cortical atrophy and a discrete underlying pathology (Mesulam, 2001). Over 50% of nfvPPA patients have FTLD tauopathy (including Pick’s disease, Corticobasal Degeneration, Progressive Supranuclear Palsy), and around 20% of patients have TDP-43 proteinopathy (predominantly type A). Conversely, the majority of svPPA has a TDP-43 pathology (Harris and Jones, 2014). Patients with svPPA present loss of word knowledge with difficulties in single-word comprehension and naming, despite relatively preserved grammar and fluency. Conversely, patients with nfvPPA show abnormality of grammar in spoken or written language, and/or apraxia of speech with relatively preserved single-word comprehension (Mesulam, 1982; Ash et al., 2010; Wilson et al., 2010; Gorno-Tempini et al., 2011).

In the past years, several studies have investigated the brain damage associated with PPA, reveling an asymmetric prominence of grey and white matter changes, neuronal loss and disease-specific proteinopathy (Grossman et al., 2008; Mesulam et al., 2008, 2014; Grossman, 2012). Concerning neuroimaging investigations, patients with svPPA show a predominant left-sided temporal patter of gray matter atrophy, with subcortical compromission of the amygdala and hippocampus (Brambati et al., 2009; Collins et al., 2017; Nigro et al., 2021). Conversely, a prominent atrophy of left frontal regions is usually observed in nfvPPA patients (Gorno-Tempini et al., 2011; Mesulam et al., 2014; Mandelli et al., 2016a,b). Moreover, studies that use diffusion tensor imaging (DTI) highlight a severe damage of white matter tracts with a lateralization involving the left inferior longitudinal fasciculus (ILF), uncinate fasciculus (UF) and posterior cingulate in svPPA patients (Galantucci et al., 2011; Agosta et al., 2012; Mahoney et al., 2013; Mesulam et al., 2014; Vandenberghe, 2016; Bouchard et al., 2019; Reyes et al., 2019) while a compromission of the left intrafrontal and frontostriatal fascicules has been reported in nfvPPA patients (Mandelli et al., 2014, 2016a).

In recent years, several studies have highlighted the potentialities of radiomics in the clinical and diagnostic work-up of patients with neurodegenerative diseases as an approach that allows capturing from radiological images non-trivial and complex features (compared to classical morphological approaches) associated with clinical and biological outcomes (Abbasian Ardakani et al., 2022). The radiomic-based analytical framework combines features of global and local regions with machine and deep learning algorithms aiming at unveiling higher-order information underlying specific disorders (Wu et al., 2019; Xiao et al., 2019; Cao et al., 2020; Feng and Ding, 2020; Liu et al., 2020; Jain et al., 2021; Kim et al., 2021; Zhou et al., 2021; Cheung et al., 2022). Studies that used radiomics on gray matter and white matter regions in patients with Alzheimer’s (AD) and Parkinson’s diseases (PD) showed promising results in terms of patients’ characterization by merging the high-throughput extraction of pattern-based information over specific brain regions with clinical data. In the field of FTLD dementias, radiomics has shown excellent performances in distinguishing FTD subtypes (Tafuri et al., 2022).

Given previous evidences of an asymmetrical brain involvement in PPA patients, in this study we applied radiomic analysis to explore white matter laterality pattern in patients with PPA variants and to investigate its relationship with performance at linguistic tasks.

## 2. Methods

### 2.1. Participants

Data used in the preparation of this retrospective study were obtained from the Frontotemporal Lobar Degeneration Neuroimaging Initiative (FTLDNI) database (for up-to-date information on participation and protocol, please visit^1^). We included 56 patients with PPA (31 svPPA and 25 nfvPPA) and 53 age- and sex-matched healthy control (HC) who had a valid baseline T1-weighted MRI sequence. In order to avoid potential bias derived from different imaging protocols, we selected exclusively images acquired at the University of California, San Francisco (i.e., the largest recruiting center). Approval for the FTLDNI protocol has been granted by institutional review board at the study site. All patients underwent clinical, imaging, language, and neuropsychological examination and fulfilled the current diagnostic criteria for PPA (Gorno-Tempini et al., 2011). The control group consisted of individuals with no previous history of diagnosed neurological or psychiatric disorder and no complaint of memory decline (more information at^2^).

### 2.2. Clinical and language assessment

The Clinical Dementia Rating scale (CDR), with its language subscore (CDR language), was administered to assess the global cognitive status (Morris, 1993; Knopman et al., 2008).

Linguistic abilities were tested with the semantic verbal fluency (animal) and the phonemic verbal fluency (d words) tests (Benton, 1969). Semantic fluency test assesses the ability to verbally generate as many words as possible from a given semantic category. In phonemic task participants are asked to produce as many words as possible beginning with the letter D in 1 minute. Finally, the total Boston Naming Test (BNT) correct score was computed as a measure of general linguistic ability, object naming and word retrieval (Kaplan et al., 1983).

### 2.3. MRI data processing

MR images were acquired on a 3 T Siemens Trio Tim system equipped with a 12-channel head coil including whole-brain three-dimensional T1 MPRAGE (TR/TE = 2,300/2.9 ms, matrix = 240 ×  256 × 160, isotropic voxels 1 mm^3^, slice thickness = 1 mm). An experienced neuroradiologist examined the images for brain abnormalities other than atrophy.

The MRI were processed with FreeSurfer 6.0 with the standard cross-sectional pipeline (*recon-all*). The pre-processing steps comprised removal of non-brain tissue, bias correction, and segmentation into gray matter (GM), white matter (WM), and cerebrospinal fluid. The brain extracted non-uniform intensity corrected image (*nu.mgz*) was used to compute the radiomics features in WM regions. In particular, the FreeSurfer white matter parcellation approach (Salat et al., 2009) was used to classify the white matter according to the nearest cortical region (Desikan et al., 2006) and obtaining 34 regions of interest (ROIs) for each hemisphere. Detailed information on these procedures have been described in previous publications (Dale et al., 1999; Fischl et al., 2002, 2004).

### 2.4. Radiomics asymmetry index

The python package PyRadiomics (van Griethuysen et al., 2017) was used to extract radiomics features from 68 white matter ROIs (34 regions for each hemisphere). For each ROI, we defined a set of 86 radiomic features, including 16 first-order features to describe voxel intensity distribution within image mask and 70 second-order textural measures to highlight spatial distribution of voxels through five different matrices: 24 features from Gray Level Co-occurrence Matrices (GLCM), 16 from Gray Level Run Length Matrices (GLRLM), 14 measures from Gray Level Dependence Matrices (GLDM) and 16 features from Gray Level Size Zone Matrices (GLSZM) (Zwanenburg et al., 2020).

Given a brain region, the Asymmetry Index (AI) was computed for each radiomics measure as the ratio of the absolute difference between left and right feature values to its sum, multiplied by 100 (Pedraza et al., 2004; Heckemann et al., 2011; Kim et al., 2012; Okada et al., 2016; Sarica et al., 2018):


$$x_{AI}=\frac{\left|x_{\text{Left}}-x_{\text{Right}}\right|}{x_{\text{Left}}+x_{\text{Right}}}*100$$

### 2.5. Statistical analysis

Data were explored with descriptive statistics (mean ± standard deviation or frequency).

Group differences in demographical, clinical data and performance at linguistic tasks were analyzed with chi-square test and Kruskal-Wallis analysis of variance followed by post-hoc test.

For each radiomics feature, analysis of variance was used to compare asymmetry index values between groups while controlling for age, gender and education, followed by effect size (Cohen’s *d*) computation (Cohen, 1988). Significance threshold was set at *p* < *0.05* after Bonferroni correction for multiple comparisons.

Partial correlation (Spearman’s correlation) was used to explore the relationship between linguistic tests performances and AI values while controlling for the influence of age, gender, education and CDR total score. We considered only moderate correlations (Spearman *r* > |0.4|) (Schober et al., 2018). Statistical analysis was performed by using R software (Version 3.6.3: R Foundation for Statistical Computing, Vienna, Austria).

White matter areas that were statistically significant in pairwise comparisons and correlation analyses were mapped onto the JHU WM tractography atlas (Wakana et al., 2007) to identify white matter tracts involved in PPA neurodegeneration.

## 3. Results

### 3.1. Participants characteristics

Demographic and clinical data are reported in Table 1. No significant group differences emerged in education and gender. Patients with nfvPPA were older than controls and svPPA patients (*p* < 0.01, Bonferroni corrected). Concerning clinical data, PPA groups had higher CDR scores than controls (value of *p* < 0.001, Bonferroni corrected). Linguistic tests scores were lower in PPA patients respect to control group (*p* < 0.001, Bonferroni corrected). Significant differences in phonemic verbal fluency test emerged between patients’ groups, with nfvPPA patients presenting lower scores than svPPA (*d words*, *p* = 0.01, Bonferroni corrected) and svPPA patients showing lower scores at BNT test than nfvPPA patients (*p* < 0.001, Bonferroni corrected).

**Table 1:** Demographic and clinical data reported as mean and standard deviation (SD) values.

	HC n = 53	nfvPPA *n* = 25	svPPA *n* = 31	Kruskal–Wallis test	Post-hoc
	Mean (SD or %)	Mean (SD or %)	Mean (SD or %)	*p*	
Age, years	64.11 (6.33)	68.56 (7.38)	62.81 (6.47)	<0.005	nfvPPAvsHC, svPPA, 0.01
Female	23 (43.4)	11 (44.0)	18 (58.1)	0.393	-
Education, years	21.96 (19.13)	15.80 (2.60)	19.06 (15.08)	0.263	-
CDR	0.03 (0.12)^a^	0.44 (0.34)	0.65 (0.32)	<0.001	HCvsnfvPPA, svPPA, <0.001
CDR language	0.0 (0.0)^a^	1.25 (0.77)	0.95 (0.51)	<0.001	HCvsnfvPPA, svPPA, <0.001
Verbal Fluency-Animal	23.53 (5.14)	11.88 (8.69)	9.03 (4.15)	<0.001	HCvsnfvPPA, svPPA, <0.001
Verbal Fluency-d words	15.87 (4.13)	6.60 (6.18)	8.97 (4.48)	<0.001	HCvsnfvPPA, svPPA, <0.001 nfvPPA vs. svPPA, 0.01
Boston Naming Test	14.32 (0.89)	12.52 (2.45)	5.71 (3.53)	<0.001	HCvsnfvPPA, svPPA, <0.001 nfvPPA vs. svPPA, <0.001

HC, Healthy Controls; nfvPPA, non-fluent variant of Primary Progressive Aphasia; svPPA, semantic variant of Primary Progressive Aphasia; CDR, Clinical Dementia Rating scale.

a Data available for 24 out of 53 healthy controls.

### 3.2. Radiomics asymmetry index analysis

**Figure 1:**
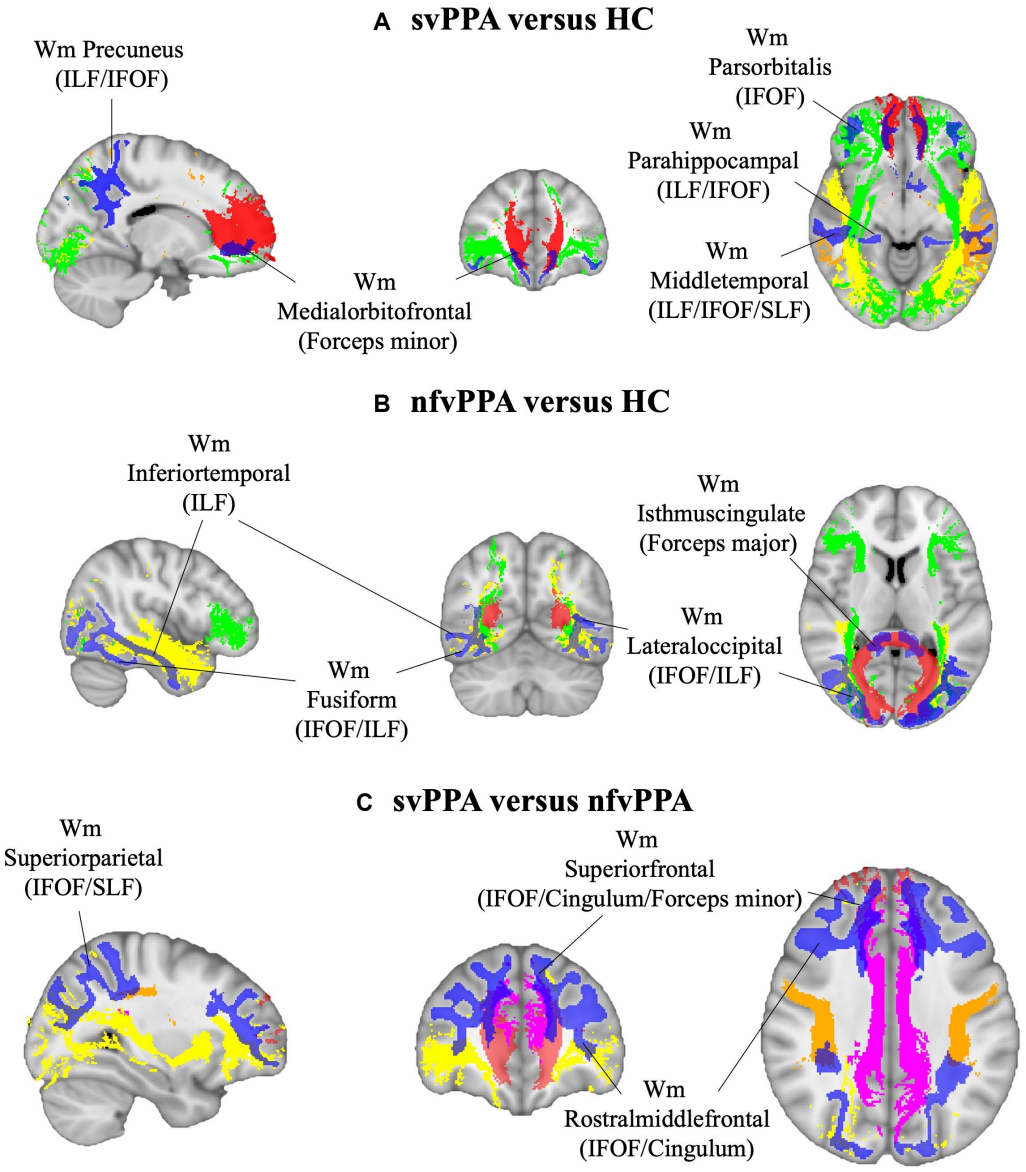
White matter ROIs (in blue) and tracts involved (red, Fornix; green, IFOF; yellow, ILF; orange, SLF; pink, cingulum) for **(A)** svPPA versus HC, **(B)** nfvPPA versus HC and **(C)** svPPA versus nfvPPA comparisons. HC, Healthy Controls; nfvPPA, non-fluent variant of Primary Progressive Aphasia; svPPA, semantic variant of Primary Progressive Aphasia; WM, white matter; IFOF, inferior fronto-occipital fasciculus; ILF, inferior longitudinal fasciculus; SLF, superior longitudinal fasciculus.

**Figure 2:**
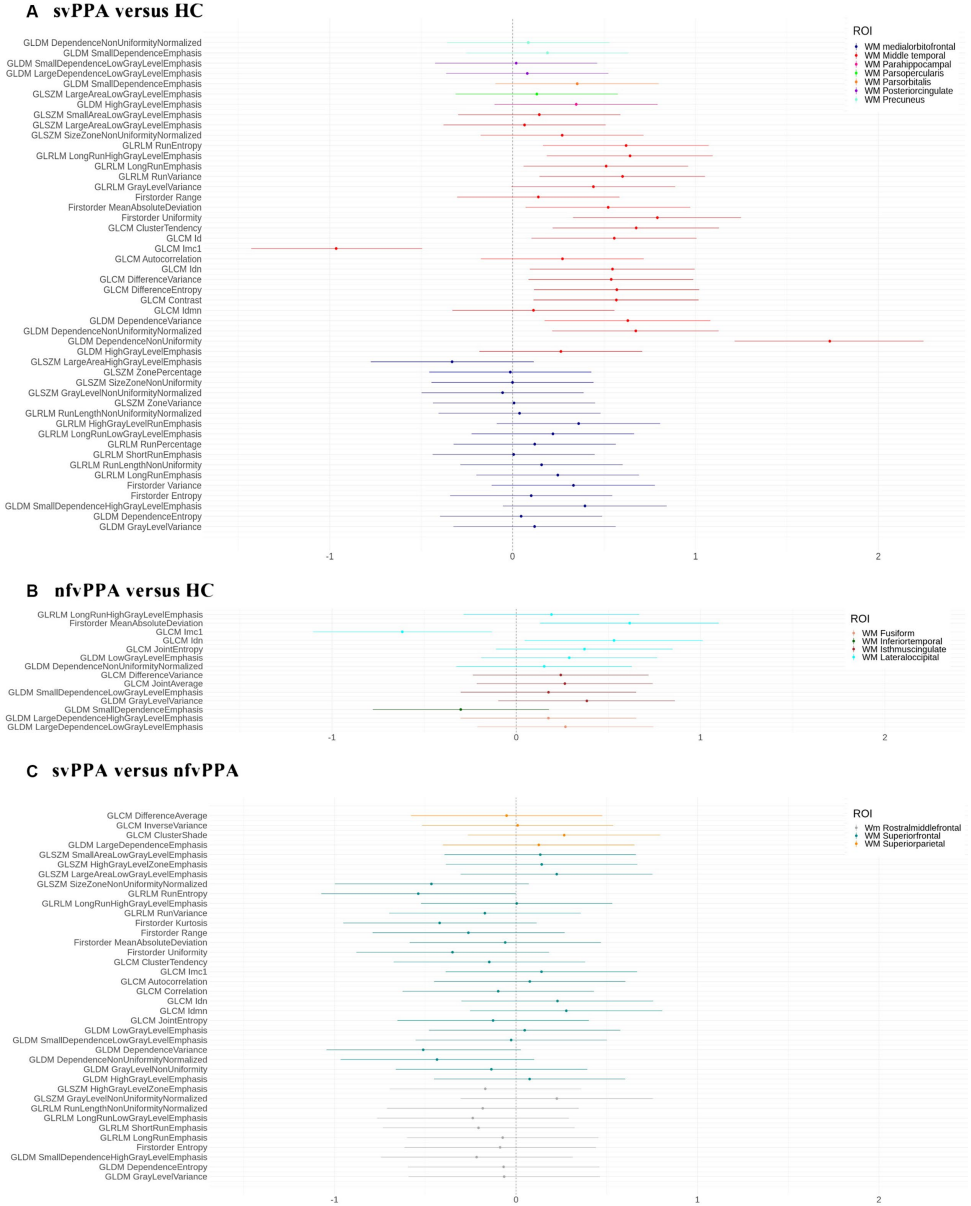
Effect size d for each binary comparison. **(A)** svPPA versus HC; **(B)** nfvPPA versus HC; **(C)** svPPA versus nfvPPA. We report the selected radiomics features (*y*-axis) for each ROI (white matter regions). HC, Healthy Controls; nfvPPA, non-fluent variant of Primary Progressive Aphasia; svPPA, semantic variant of Primary Progressive Aphasia; WM, white matter; GLCM, Gray Level Co-occurrence Matrices; GLRLM, Gray Level Run Length Matrices; GLDM, Gray Level Dependence Matrices; GLSZM, Gray Level Size Zone Matrices.

As reported in Figure 1, svPPA and nfvPPA patients showed an asymmetric pattern of compromission involving ILF and IFOF white matter fasciculi. In particular, radiomics asymmetry in svPPA patients mostly involved white matter regions adjacent to the medial orbitofrontal and middle temporal cortices as part of ILF/IFOF, SLF fasciculi and forceps minor white matter tract (detailed radiomics features are reported in Figure 2A). On the contrary, nfvPPA showed an asymmetrical radiomics in fusiform, isthmus cingulate and lateral occipital regions comprised in ILF, IFOF and forceps major fiber tracts (Figure 2B). Finally, between groups comparison depicted significative lateralized measures in rostral middle frontal, superior parietal and superior frontal cortices, corresponding to SLF, IFOF, cingulum and forceps minor white matter tracts (Figures 1C, 2C).

### 3.3. Correlation analysis

Results of correlation analysis are reported in Table 2. In nfvPPA patients, a significant correlation (*p < 0.0059*, *r = 0.592*) was found between *semantic fluency* scores and AI values of GLCM lmc1 (Informational measure of correlation-1, for more details visits^3^) evaluated in WM region adjacent to lateral occipital gyrus (ILF/IFOF). On the other hand, BNT scores were significantly associated with two AI measures in WM near the middle temporal (ILF/IFOF/SLF) (*p* = 0.0002, *r* = 0.654) and parahippocampal gyri (ILF/IFOF) (*p* = 0.0004, *r* = −0.631) of svPPA patients, namely a positive correlation with the range of voxel intensities and a negative correlation with GLDM high grey level emphasis values. No significant correlation was found between AI values and *phonemic fluency* for both PPA groups.

**Table 2:** Spearman’s correlation coefficients of Verbal Fluency-Animal and Boston Naming test scores with radiomics AI for nfvPPA and svPPA patients.

		svPPA		nfvPPA	
Asymmetry index	ROI	Value of *p*	*r*	Value of *p*	*r*
Verbal Fluency—Animal					
GLDM SmallDependenceEmphasis	WM precuneus (ILF/IFOF)	0.292	−0.210	0.0475	−0.448
GLDM HighGrayLevelEmphasis	WM parahippocampal (ILF/IFOF)	0.007	−0.503	0.9288	0.021
GLCM lmc1	WM lateraloccipital (ILF/IFOF)	0.548	0.121	0.0059	0.592
Boston Naming Test					
GLRLM RunEntropy	WM middletemporal (ILF/IFOF/SLF)	0.055	−0.373	0.017	−0.523
GLDM SmallDependenceLowGrayLevelEmphasis	WM posteriorcingulate (Cingulum)	0.626	0.098	0.003	−0.617
GLCM Imc1	WM lateraloccipital (ILF/IFOF)	0.730	0.069	0.012	0.544
GLDM DependenceNonUniformity	WM middletemporal (ILF/IFOF/SLF)	0.091	−0.331	0.053	−0.437
Firstorder Range	WM middletemporal (ILF/IFOF/SLF)	0.0002	0.654	0.242	0.273
GLDM HighGrayLevelEmphasis	WM parahippocampal (ILF/IFOF)	0.0004	−0.631	0.449	−0.179
GLCM ClusterTendency	WM middletemporal (ILF/IFOF/SLF)	0.001	−0.580	0.104	0.374
Firstorder Uniformity	WM middletemporal (ILF/IFOF/SLF)	0.005	−0.525	0.758	−0.073
GLDM DependenceNonUniformityNormalized	WM middletemporal (ILF/IFOF/SLF)	0.025	−0.430	0.344	−0.223
GLDM SmallDependenceEmphasis	WM precuneus (ILF/IFOF)	0.019	−0.448	0.189	−0.305

nfvPPA, non-fluent variant of Primary Progressive Aphasia; svPPA, semantic variant of Primary Progressive Aphasia; WM, white matter; GLCM, Gray Level Co-occurrence Matrix; GLRLM, Gray Level Run Length Matrix; GLDM, Gray Level Dependence Matrix; WM, white matter; IFOF, inferior fronto-occipital fasciculus; ILF, inferior longitudinal fasciculus; SLF, superior longitudinal fasciculus.

## 4. Discussion

In the study, we investigated the asymmetry of radiomics features in white matter of svPPA and nfvPPA patients. Compared to controls, PPA patients were characterized by a lateralized structural compromission involving fronto-temporal and occipital white matter areas corresponding to different fiber tracts, namely the inferior longitudinal (ILF) and superior longitudinal (SLF) fasciculi, inferior fronto-occipital fasciculus (IFOF), fornix and cingulum (Routier et al., 2018; Cocquyt et al., 2020). Moreover, an increased asymmetry of specific radiomic measurements in white matter areas adjacent to the lateral occipital gyrus was associated with lower semantic fluency performance in nfvPPA.

Overall, our findings provide further evidence that PPA patients exhibit an asymmetric damage that involved both gray and white matter morphology. Concerning svPPA patients, radiomics findings reported several significative AIs in ILF/IFOF and SLF with a predominance of the white matter near the middle temporal gyrus. Respect to previous study (Lombardi et al., 2021), our finding was in line with the left-sided compromission of this region. Similarly, tractography studies on diffusion-weighted imaging data also confirmed this lateralized damage (Agosta et al., 2012; Routier et al., 2018). In particular, tract analysis showed a focal asymmetric reduction of fractal anisotropy involving the left temporal regions (left SLF) (Bouchard et al., 2019), corresponding to an increased mean diffusivity of ILF, bilaterally. Non fluent/agrammatic variant of PPA, instead, showed an asymmetric white matter damage close to the lateral occipital gyrus, corresponding to ILF and IFOF tracts (Galantucci et al., 2011; Mandelli et al., 2014). Structural compromission of this WM region has been reported in previous voxel-wise study, depicting a lateralized damage of inferior temporal and lateral occipital WM in nfvPPA (Lombardi et al., 2021). This finding was also confirmed by diffusion MRI results observing a fractal anisotropy decreasing in left occipital WM fibers in nfvPPA respect to healthy subjects (Agosta et al., 2012). Noteworthy, the most discriminative WM asymmetry to differentiate svPPA from nfvPPA was found in frontal regions (cingulum and the forceps minor WM tracts). Our result was suggestive of a more pronounced lateralization in frontal WM of nfvPPA respect to svPPA patients (Catani et al., 2013; Spinelli et al., 2017). Furthermore, radiomics analysis assessed the left-sided compromission of the ‘frontal aslant tract’ in nfvPPA patients, as the principal pathway that connect the frontal gyrus with supplementary and pre-supplementary motor areas and playing a crucial role in speech production (Catani et al., 2013; Mandelli et al., 2014; Canu et al., 2019).

Correlation analysis highlighted a strong relationship between the severity of linguistic deficits and AI of radiomics measures particularly between semantic fluency and asymmetry of lateral occipital white matter in nfvPPA patients. This neural correlate confirmed previous findings that demonstrated an association between semantic fluency deficit and a cortical thinning of the left lateral occipital cortex and lingual gyrus (Vonk et al., 2019). Moreover, Li et al. (2017) highlighted the same correlation results with a left-sided compromission of the IFOF tract, posteriorly starting from the lateral occipital white matter (Almairac et al., 2015). On the other hand, our lateralization analysis revealed an association in svPPA patients between WM AIs in the areas close to the parahippocampal/middle temporal and the Boston Naming Test scores. These findings further corroborate the connection of noun production and WM tracts projecting in temporal regions (including the cingulum), underlining the key role of the frontotemporal network in noun production (Ash et al., 2013). Of note, a study of surface stimulation over the parahippocampal gyrus resulted in aphasic reactions, suggesting that it has a role in language comprehension and production (Sharp et al., 2004).

Overall, the strength of our approach raised from the potentiality of radiomics to capture the asymmetric structural deterioration of white matter in PPA patients. Indeed, radiomics was able to highlight the lateralized damage of the principal tracts involved in PPA variants. We demonstrated a focal involvement of the inferior longitudinal and inferior fronto-occipital fasciculi, as part of the speech-production network. Furthermore, correlation analysis disclosed another captivating finding, highlighting the importance of ILF and IFOF tracts as involved in written and spoken language production. Accordingly, radiomics asymmetry may represent a candidate severity marker for language impairments in PPA patients. Overall, all these findings suggest our novel approach extracted from structural MRI makes the proposed study noteworthy and easily reproducible in the clinical practice routine as support to diagnosis and prognosis steps.

The principal limitation of our work is the lack of confirmation of the lateralization of white matter with diffusion-based data. Future studies should merge radiomics approach with diffusion weighted images to investigate the asymmetry of tract damage and to highlight possible association with language deficits in PPA variants. Moreover, the study has a relatively small sample size, which may not be entirely representative of the PPA spectrum, also lacking patients with logopenic variant. Of note, none of our patients had a histopathological diagnostic confirmation. Finally, the lack of clinical interpretation of the selected radiomic measurements makes it difficult to accurately interpret the results. Future studies should points out possible correlations of radiomics with biological data (genomics, transcriptomics, metabolomics, etc.) to give further insights into the clinical relevance of the radiomic findings.

## 5. Conclusion

This work provided important findings over structural white matter asymmetry in Primary Progressive Aphasia. Radiomics features depicted a mostly lateralized pattern on semantic variant respect to non-fluent/agrammatic PPA patients involving damage of principal linguistic fiber tracts, respectively the temporal and occipital parts of inferior longitudinal/fronto-occipital fasciculi. Moreover, the evaluation of radiomics asymmetry in association with neuropsychological fluency tasks highlighted novel neuroanatomical correlates with semantic fluency and Boston Naming Test specific for each variant of PPA.

## Data availability statement

The original contributions presented in the study are included in the article, further inquiries can be directed to the corresponding author.

## Ethics statement

Ethical review and approval was not required for the study on human participants in accordance with the local legislation and institutional requirements. Written informed consent for participation was not required for this study in accordance with the national legislation and the institutional requirements.

## Author contributions

BT, SN, and GL contributed to conception and design of the study. BT, SN, MF, DU, VG, and RB contributed to analysis and interpretation of data. BT drafted the article. SN, DU, MF, RB, VG, and GL contributed to revise it critically for important intellectual content. BT, SN, MF, DU, RB, VG, and GL provided approval for publication of the version to be published and agreed to be accountable for all aspects of the work in ensuring that questions related to the accuracy or integrity of any part of the work are appropriately investigated and resolved. All authors contributed to the article and approved the submitted version.

## Funding

The FTLDNI was funded through the National Institute of Aging and started in 2010. The primary goals of FTLDNI are to identify neuroimaging modalities and methods of analysis for tracking frontotemporal lobar degeneration and to assess the value of imaging vs. other markers in diagnostic roles. The principal investigator of NIFD was Howard Rosen, MD, at the University of California, San Francisco. The data are the result of collaborative efforts at three different sites in North America. Access to the FTLDNI data was approved by the data access committee. This work has been supported with the founding of Regione Puglia and CNR for Tecnopolo per la Medicina di Precisione. D.G.R. n. 2117 of 21.11.2018 (CUPB84I18000540002)—C.I.R.E.M.I.C. (Research Center of Excellence for Neurodegenerative Diseases and Brain Aging)—University of Bari “Aldo Moro.” Data collection and sharing for this project was funded by the Frontotemporal Lobar Degeneration Neuroimaging Initiative (National Institutes of Health Grant R01AG032306). The study is coordinated through the University of California, San Francisco, Memory and Aging Center. FTLDNI data are disseminated by the Laboratory for Neuro Imaging at the University of Southern California.

## Conflict of interest

The authors declare that the research was conducted in the absence of any commercial or financial relationships that could be construed as a potential conflict of interest.

## Publisher’s note

All claims expressed in this article are solely those of the authors and do not necessarily represent those of their affiliated organizations, or those of the publisher, the editors and the reviewers. Any product that may be evaluated in this article, or claim that may be made by its manufacturer, is not guaranteed or endorsed by the publisher.

## Footnotes

^1^ http://memory.ucsf.edu/research

^2^ https://memory.ucsf.edu/research-trials/research/4rtni-2

^3^ https://pyradiomics.readthedocs.io/en/latest/features.html
